# Foot Placement Modification for a Biped Humanoid Robot with Narrow Feet

**DOI:** 10.1155/2014/259570

**Published:** 2014-01-27

**Authors:** Kenji Hashimoto, Kentaro Hattori, Takuya Otani, Hun-Ok Lim, Atsuo Takanishi

**Affiliations:** ^1^Waseda Research Institute for Science and Engineering, Waseda University, No. 41-304, 17 Kikui-cho, Shinjuku-ku, Tokyo 162-0044, Japan; ^2^Graduate School of Science and Engineering, Waseda University, No. 41-304, 17 Kikui-cho, Shinjuku-ku, Tokyo 162-0044, Japan; ^3^Faculty of Engineering, Kanagawa University, 3-27-1 Rokkakubashi, Kanagawa-ku, Yokohama 221-8686, Japan; ^4^Humanoid Robotics Institute (HRI), Waseda University, 2-2 Wakamatsu-cho, Shinjuku-ku, Tokyo 162-8480, Japan; ^5^Department of Modern Mechanical Engineering, Waseda University, 2-2 Wakamatsu-cho, Shinjuku-ku, Tokyo 162-8480, Japan

## Abstract

This paper describes a walking stabilization control for a biped humanoid robot with narrow feet. Most humanoid robots have larger feet than human beings to maintain their stability during walking. If robot's feet are as narrow as humans, it is difficult to realize a stable walk by using conventional stabilization controls. The proposed control modifies a foot placement according to the robot's attitude angle. If a robot tends to fall down, a foot angle is modified about the roll axis so that a swing foot contacts the ground horizontally. And a foot-landing point is also changed laterally to inhibit the robot from falling to the outside. To reduce a foot-landing impact, a virtual compliance control is applied to the vertical axis and the roll and pitch axes of the foot. Verification of the proposed method is conducted through experiments with a biped humanoid robot WABIAN-2R. WABIAN-2R realized a knee-bended walking with 30 mm breadth feet. Moreover, WABIAN-2R mounted on a human-like foot mechanism mimicking a human's foot arch structure realized a stable walking with the knee-stretched, heel-contact, and toe-off motion.

## 1. Introduction

**Figure 1:**
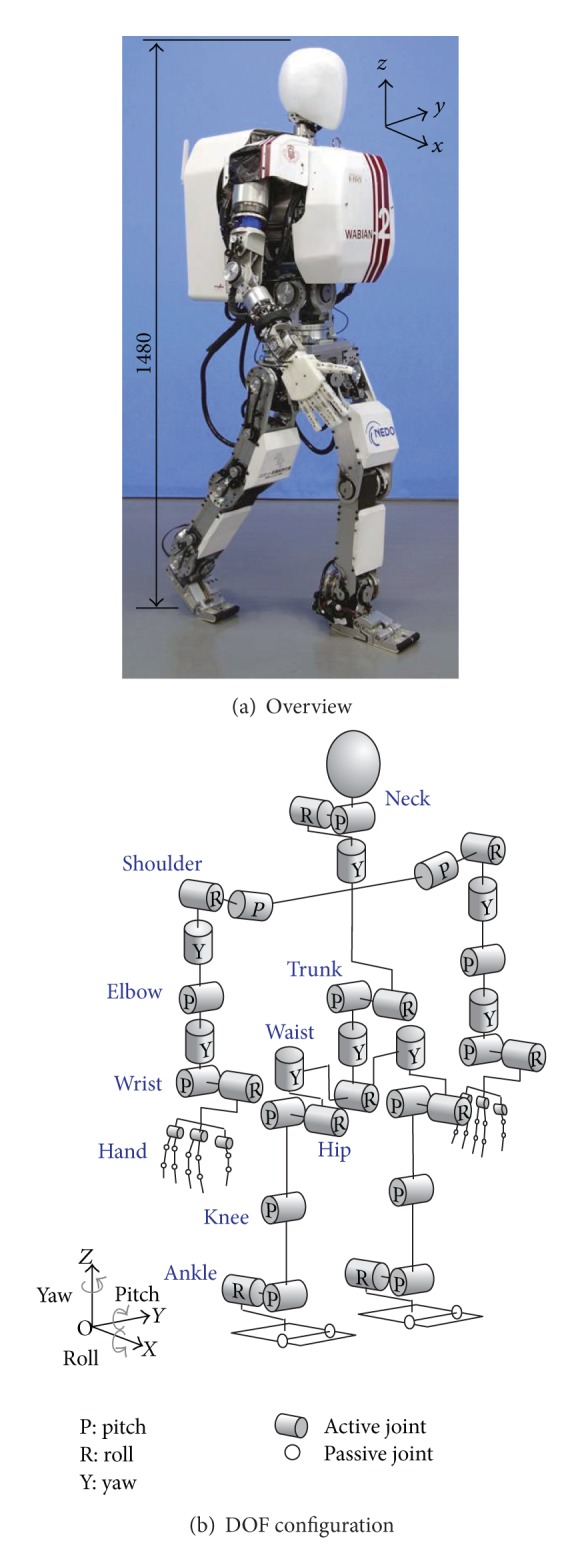
WABIAN-2R (Waseda Bipedal Humanoid—No. 2 Refined).

We have developed a biped humanoid robot named WABIAN-2R (Waseda Bipedal Humanoid—No. 2 Refined) as a human motion simulator to mimic human's motions and mechanisms [1] (see Figure 1). Its height is 1480 mm with 63.8 kg weight, and it has 41 degrees of freedom. So far, WABIAN-2R has realized a stretch-knee gait by utilizing a 2-DOF (Roll, Yaw) waist mimicking a human's pelvic movement. WABIAN-2R has also achieved a human-like walk with the knee-stretched, heel-contact, and toe-off motion by utilizing a foot mechanism with a passive toe joint [2]. But the foot breadth is 160 mm and wider than human beings. The average foot breadth of humans is as narrow as 90 mm [3].

Most of the full size humanoid robots such as ASIMO [4], Toyota running robot [5], HRP-2 [6], HRP-3 [7], Johnnie [8], and HUBO [9] have larger feet than human beings to maintain stability during walking. CB-i [10] has as narrow feet as humans, but it has not yet realized a stable dynamic walking. Recently, HRP-4C [11, 12] realized a human-like walking, but it focuses on single toe support and has not realized the heel contact on the ground. So far, there are few biped robots which have realized a human-like walking with knee-stretched, heel-contact, and toe-off motion with narrow feet.

**Figure 2:**
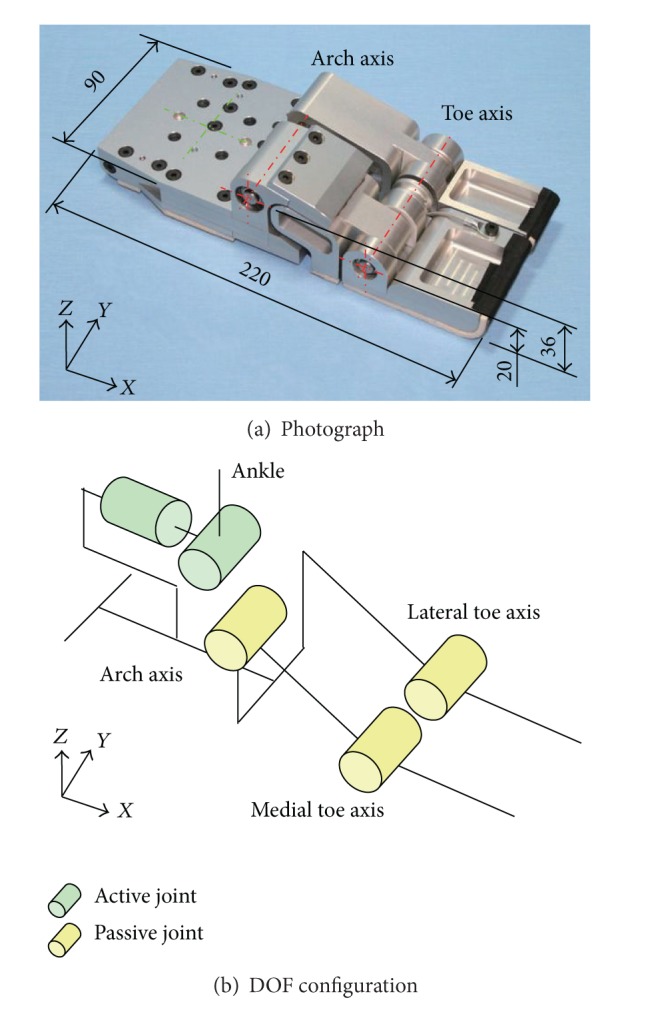
Human-like foot mechanism with the medial longitudinal arch.

Meanwhile, we have developed a new biped foot mechanism mimicking a human's foot arch structure as shown in Figure 2 [13], and the foot breadth is as narrow as 90 mm like humans. WABIAN-2R became unstable with conventional stabilization controls when the foot breadth becomes 100 mm or narrower, and WABIAN-2R with the human-like foot mechanism tends to fall down laterally because the foot breadth is shorter than the foot length. Therefore, we considered that biped walking stability can be improved by changing lateral foot placement.

There are some previous researches on biped stabilization by changing foot placement [14, 15], but most of them assume that the foot contacts the ground at a point. Miura and Shimoyama [14] have developed the BIPER series which can perform a dynamically stable walk with suitable control, but they are statically unstable due to the assumption described above. Townsend [15] proposed the biped stabilization through lateral foot placement based on conditions at the onset of each new step. The effectiveness of the proposed method was validated through simulations, but it is difficult to apply the controller to a real biped robot whose feet have a certain amount of areas, although it can work well if robot's feet contact the ground at a point. If robot's feet do not contact the ground at a point, the effect of areas on the feet must be considered to realize a dynamic walk.

In this paper, we aim to develop a walking stabilization control for a biped humanoid robot with narrow feet and realize a stable walking with the knee-stretched, heel-contact, and toe-off motion. The proposed control stabilizes a robot by modifying a foot placement according to the robot's attitude angle. This paper is organized as follows.  Section 2 describes the details of a new stabilization control. In Section 3, experimental results are shown.  Section 4 provides conclusions and future work.

## 2. Foot Placement Modification

**Figure 3:**
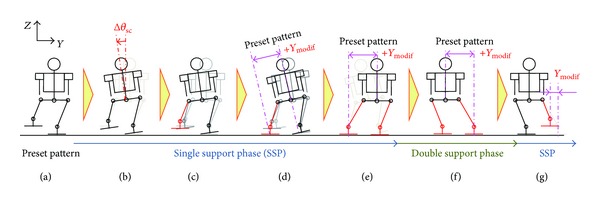
Outline of the foot placement modification control.

The proposed control stabilizes a biped robot by modifying a foot placement according to an attitude angle of the robot. Specifically, it modifies a foot angle about the roll axis and a foot-landing point along the lateral axis so that a swing foot contacts the ground horizontally. This control consists of the following five key points:

- modification of a foot placement according to an attitude angle (Figures 3(b) and 3(c));
- virtual compliance control to reduce a foot-landing impact (Figure 3(d));
- returning motion of the landing foot to a preset pattern along the vertical axis and about the roll and pitch axes (Figure 3(e));
- modification of a waist trajectory considering a foot-landing point variation during double support phase (Figure 3(f));
- returning motion of a swing leg along the lateral axis considering the convergence of a foot-landing point variation (Figure 3(g)).

**Figure 4:**
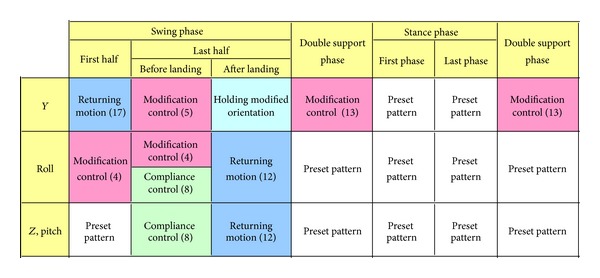
Timing chart of the foot placement modification control.

**Figure 5:**
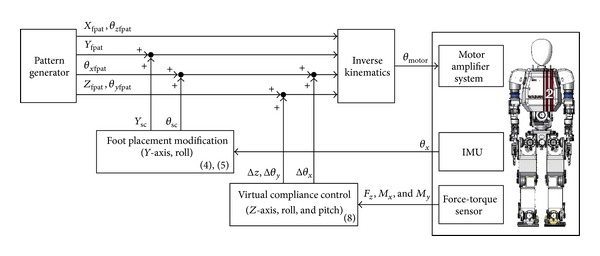
Block diagram of the foot placement modification control. *X*_fpat_, *Y*_fpat_, and *Z*_fpat_ are preset foot positions along the *x*-, *y*-, and *z*-axes respectively. *θ*_*x*fpat_, *θ*_*y*fpat_, and *θ*_*z*fpat_ are preset foot orientations about the roll, pitch, and yaw axes, respectively. *θ*_*x*_ is the roll attitude angle, and *F*_*z*_, *M*_*x*_, and *M*_*y*_ are ground reaction forces in the *z*, roll, and pitch directions.

Figures 3, 4, and 5 depict the outline, timing chart, and block diagram of the proposed control, respectively. This control serves to maintain mechanical energy at the landing, which means that the phase portrait of the proposed system should depict a closed curve. If the mechanical energy diverges, the phase portrait depicts a hyperbolic curve, and a stable walk cannot be maintained.

### 2.1. Foot Placement Modification according to an Attitude Angle

An inertial measurement unit (IMU) is mounted on a robot's trunk, and an attitude angle is estimated, how much a robot falls to the inside. According to the attitude angle, a foot angle is modified about the roll axis so that a swing foot contacts the ground horizontally. A foot-landing point is also changed laterally to inhibit the robot from falling to the outside.

The modification of a swing foot's angle is introduced during a swing phase. The data of the attitude sensor are updated every 10 ms, and a quintic polynomial is used to modify the foot angle as follows:

(1) θsc(t)=θsc(t−tsc)+Δθsc·f(s1Tsc),

(2) f(s1Tsc)=a1(s1Tsc)5+b1(s1Tsc)4+c1(s1Tsc)3          +d1(s1Tsc)2+e1·s1Tsc+f1,

where *θ*_sc_(*t*) is the modification angle of a swing foot about the roll axis, *T*_sc_ is the modification time of 10 ms, *t*_sc_ is the time during a specified time *T*_sc_, and Δ*θ*_sc_ is the angle difference between *θ*_sc_(*t*) and the current attitude angle when *t*_sc_ = 0.

To calculate quantic polynomial coefficients, boundary conditions are given as follows:

(3) f(0Tsc)=0,  f′(0Tsc)=0,  f′′(0Tsc)=0,f(TscTsc)=1,  f′(TscTsc)=0,  f′′(TscTsc)=0.

Coefficients are obtained, and (1) is expressed as follows:

(4) θsc(t)=θsc(t−tsc) +Δθsc{6(tscTsc)5−15(tscTsc)4+10(tscTsc)3}.

The modification of a foot-landing point is introduced during the last half of a swing phase. The swing leg is opened outward in proportion to the roll angle of the robot's trunk. A quintic polynomial is also used to modify the foot-landing position as follows:

(5) Ysc(t)=Ysc(t−tsc) +ΔYsc{6(tscTsc)5−15(tscTsc)4+10(tscTsc)3},ΔYsc=KY·Δθsc−Ysc(t−tsc),

where *Y*_sc_(*t*) is the modification value of a swing foot along the lateral axis and *K*_*Y*_ is a gain to determine the modification value in proportion to the angle, how much a robot falls inside. *K*_*Y*_ was experimentally determined to be 0.5 m/rad in this research.

### 2.2. Virtual Compliance Control to Reduce a Foot-Landing Impact

In this method, a foot contacts the ground earlier than planned because the robot falls inside during walking. Therefore, to reduce a foot-landing impact, we applied a virtual compliance control with only a damping component to the vertical axis and the roll and pitch axes of the foot. Consider

(6) F=CΔx˙f,

where **F** = [*F*_*z*_ *M*_*x*_ *M*_*y*_]^*T*^ is the force/moment vector acting on the compliance center of the foot, Δ**x**_*f*_ = [Δ*z* Δ*θ*_*x*_ Δ*θ*_*y*_]^*T*^ is a compliance displacement from the reference walking pattern of the foot coordinate origin, and **C** = diag⁡{*C*_*z*_, *C*_roll_, *C*_pitch_} is a virtual damping coefficient.

Foot velocity is calculated as follows:

(7) Δx˙f(t)=Δxf(t)−Δxf(t−Δt)Δt,

where Δ*t* is the control cycle of 1 ms, Δ**x**_*f*_(*t*) is a compliance displacement of the foot coordinate origin, and Δ**x**_*f*_(*t* − Δ*t*) is a compliance displacement of the foot coordinate origin before one control cycle.

Using (7), (6) is modified as follows:

(8) Δxf(t)=[CΔt]−1·F+Δxf(t−Δt).

A virtual compliance control is applied based on this equation during the last half of a swing phase.

### 2.3. Returning Motion of the Landing Foot to a Preset Pattern along the Vertical Axis and about the Roll and Pitch Axes

The modification of a foot placement is completed through the above procedures. But the motions of a biped robot should return to preset walking patterns to realize a continuous walk. After foot landing on the ground, modification values of the landing foot return to zero along the vertical axis and about the roll and pitch axes. The following quintic polynomial is used for the returning motion:

(9) θretrun(t)=θmodif·g(s2Tr),

(10) g(s2Tr)=a2(s2Tr)5+b2(s2Tr)4+c2(s2Tr)3          +d2(s2Tr)2+e2·s2Tr+f2,

where *θ*_modif_ = [*z*_modif_ *θ*_modif_*x*_ *θ*_modif_*y*_]^*T*^ is the modified value along the vertical axis and about the roll and pitch axes and *T*_*r*_ is the total time of the returning phase.

Boundary conditions are given as follows to calculate quantic polynomial coefficients:

(11) g(0Tr)=1,  g′(0Tr)=0,  g′′(0Tr)=0,g(TrTr)=0,  g′(TrTr)=0,  g′′(TrTr)=0.

Coefficients are obtained, and (9) is expressed as follows:

(12) θretrun(t)=θmodif{−6(tTr)5+15(tTr)4−10(tTr)3+1}.

### 2.4. Modification of a Waist Trajectory Considering a Foot-Landing Point Variation during Double Support Phase

During the last half of a swing phase, a swing foot position was changed laterally. Therefore, before the swing leg enters the next stance phase, a waist trajectory should be modified by the same modification value during the double support phase.

Specifically, the modification value of the foot which was a swing leg returns to zero linearly, and the same value is subtracted from the other foot during the double support phase. That is to say, a waist trajectory is modified by changing both feet positions although the distance between both feet does not change. Consider

(13) Yswing(t)={1−tTd}Ymodif,Ystance(t)=−tTdYmodif,

where *Y*_swing_(*t*) is the modification value of the foot that was a swing foot, *Y*_stance_(*t*) is the modification value of the other foot, *Y*_modif_ is the total modification value of a swing foot along the lateral axis during the last half of a swing phase, and *T*_*d*_ is the total time of the double support phase.

### 2.5. Returning Motion of a Swing Leg along the Lateral Axis Considering the Convergence of a Foot-Landing Point Variation

If the motions of a biped robot return to the preset walking pattern at every step, the trunk will incline at the same degree angle, and the same modification value will occur along the lateral axis at every step. Therefore, we consider that it is not necessary to return a swing leg to the preset pattern at every step.

The returning motion along the lateral axis is generated considering the convergence of a foot-landing point variation by comparing the variation before one step (*Y*_*m*−1_) with that before two steps (*Y*_*m*−2_). This returning motion is introduced during the first half of a swing phase.

(i) *Y*_*m*−2_ < *Y*_*m*−1_. In this case, a lateral modification value increases as the number of steps increases, and the next modification value is likely to increase. Therefore, the modified value is held without returning a swing leg to the preset walking pattern.

(ii) *Y*_*m*−2_ ≥ *Y*_*m*−1_. Because a modification value tends to converge in this case, a swing leg trajectory along the lateral axis returns to the preset walking pattern. But in principal, the modification value never becomes zero. Therefore, a returning motion, *Y*_retrun_(*t*), is generated by returning the half of the variation before one step (*Y*_*m*−1_) using a quantic polynomial as follows:

(14) Yretrun(t)=Ym−1+Ym−12·h(s3Ts),

(15) h(s3Ts)=a3(s3Ts)5+b3(s3Ts)4+c3(s3Ts)3 +d3(s3Ts)2+e3·s3Ts+f3,

where *T*_*s*_ is the total time of the first half of a swing phase.

To calculate quantic polynomial coefficients, boundary conditions are given as follows:

(16) h(0Ts)=0,  h′(0Ts)=0,  h′′(0Ts)=0,h(TsTs)=−1,  h′(TsTs)=0,  h′′(TsTs)=0.

Coefficients are obtained, and (14) is expressed as follows:

(17) Yretrun(t)=Ym−1+Ym−12 ×{−6(tTs)5+15(tTs)4−10(tTs)3}.

## 3. Experimental Tests and Consideration

To evaluate the proposed method, we implemented the developed method on a biped humanoid robot WABIAN-2R (Figure 1) and conducted walking experiments with narrow feet. We also conducted walking experiments by using WABIAN-2R mounted on a human-like foot mechanism with the medial longitudinal arch as shown in Figure 2.

### 3.1. Walking Experiments with Narrow Feet

Firstly, a narrow rubber pad is attached to the sole of the robot's feet, and we conducted walking experiments. In this experiment, WABIAN-2R walks with bending the knees.

#### 3.1.1. Narrow Feet with 90 mm Breadth

**Figure 6:**
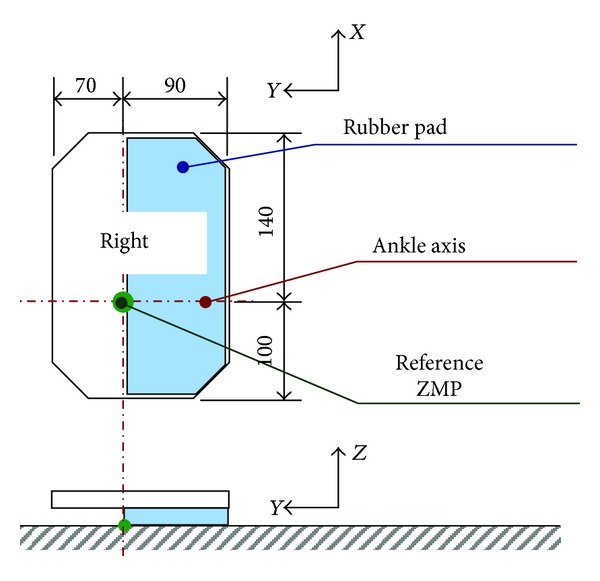
Narrow feet with 90 mm breadth. A rubber pad with 90 mm breadth is attached to the sole of the robot's feet.

A rubber pad with 90 mm breadth is attached to the sole as shown in Figure 6, which makes the robot fall down to the inside. Forward walking experiments were conducted with a step length of 200 mm/step and a walking cycle of 1.0 s/step.

**Figure 7:**
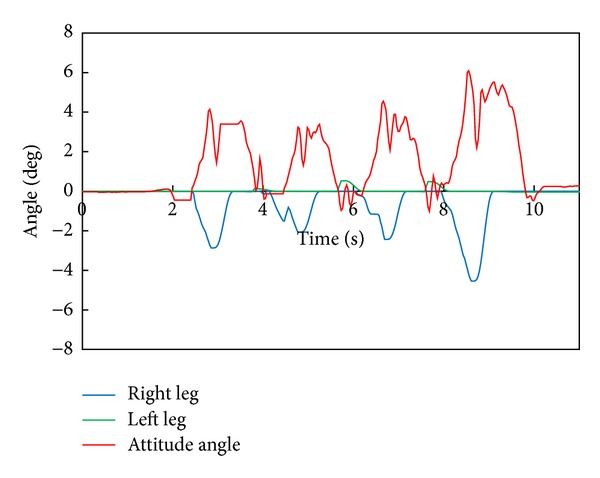
WABIAN-2R's attitude angle and the modification angle of a swing foot about the roll axis with 90 mm breadth feet.

Figure 7 depicts the robot's attitude angle and the modification angle of a swing foot about the roll axis. We can find that the robot tends to fall to the right side and the foot angle of a right leg is modified according to the attitude angle.

**Figure 8:**
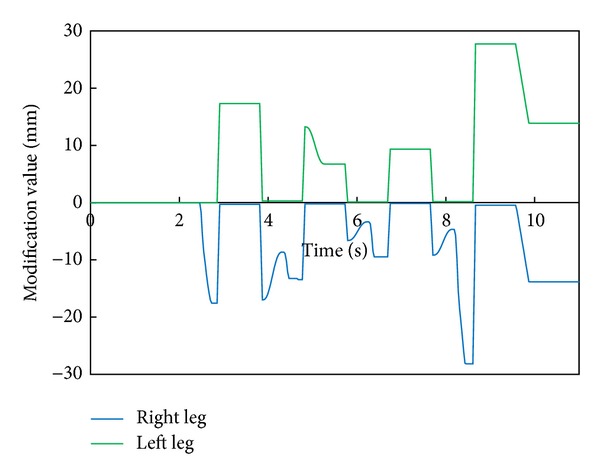
Modification value of a swing foot along the lateral axis with 90 mm breadth feet.

Figure 8 shows the modification value of a swing foot along the lateral axis. We can find that a foot-landing point is also modified according to the modified foot angle about the roll axis and a waist trajectory is modified by changing the foot positions of both legs during the double support phase.

**Figure 9:**
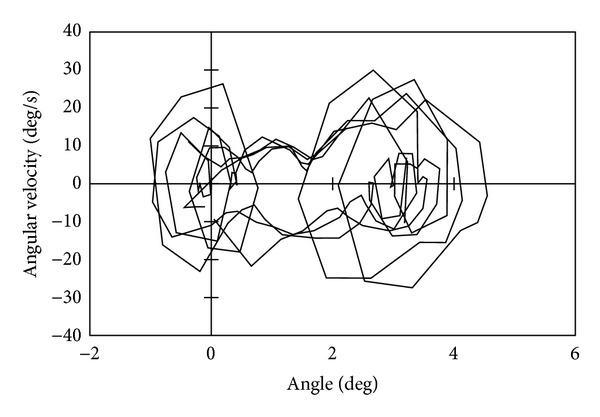
Phase portrait when walking with 90 mm breadth feet.

Figure 9 shows the phase portrait of this experiment, and we can find that the phase portrait depicts a closed curve. The circles of either end of the limit cycle are observed due to the upper body vibration caused by foot-landing impact. Without the proposed stabilization, the phase portrait did not depict a closed curve, and the robot fell down.

#### 3.1.2. Narrow Feet with 30 mm Breadth

**Figure 10:**
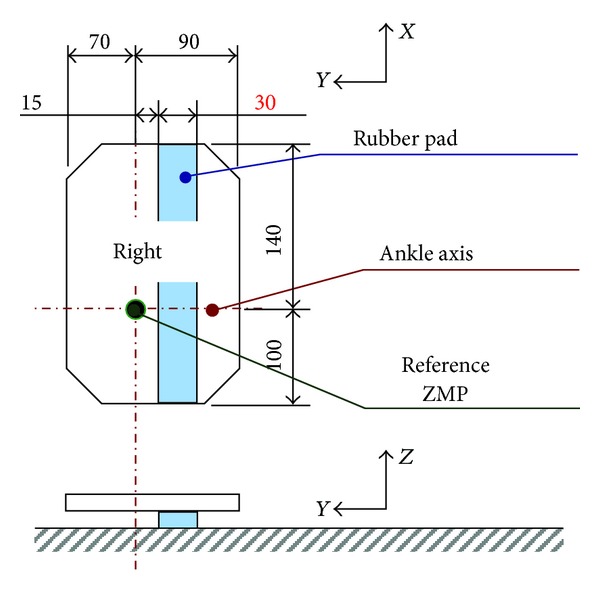
Narrow feet with 30 mm breadth. A rubber pad with 30 mm breadth is attached to the sole of the robot's feet.

To recognize the limitation of the proposed method, we conducted walking experiments with a narrower rubber pad. WABIAN-2R realized stable walks with narrow feet with 30 mm breadth. The step length was 200 mm/step and a walking cycle was 1.0 s/step. In this experiment, a rubber pad with 30 mm breadth is attached to the sole as shown in Figure 10.

**Figure 11:**
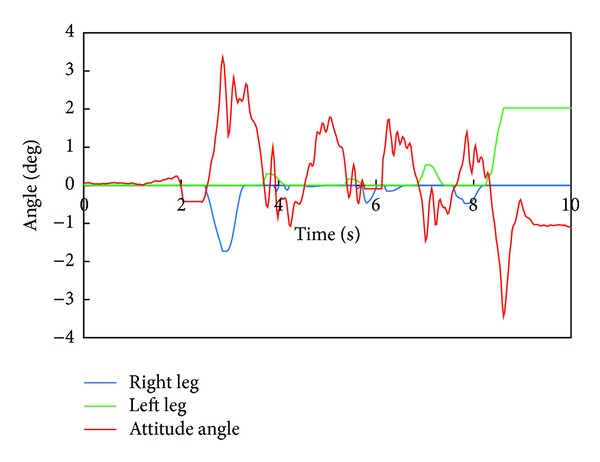
WABIAN-2R's attitude angle and the modification angle of a swing foot about the roll axis with 30 mm breadth feet.

**Figure 12:**
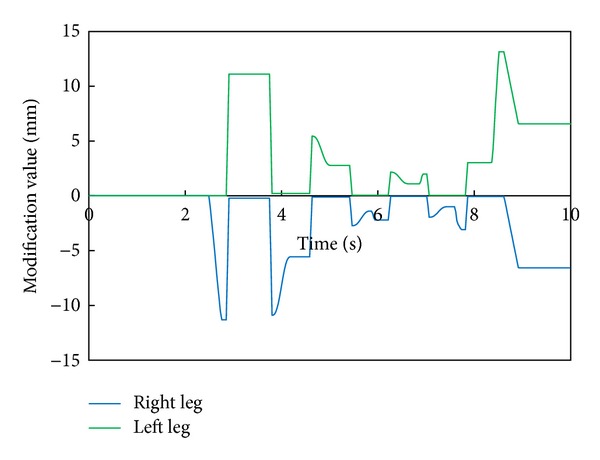
Modification value of a swing foot along the lateral axis with 30 mm breadth feet.

Figure 11 depicts the robot's attitude angle and the modification angle of a swing foot about the roll axis.  Figure 12 shows the modification value of a swing foot along the lateral axis. We can find that the foot angle and the foot-landing point of a swing leg are modified according to the attitude angle.

**Figure 13:**
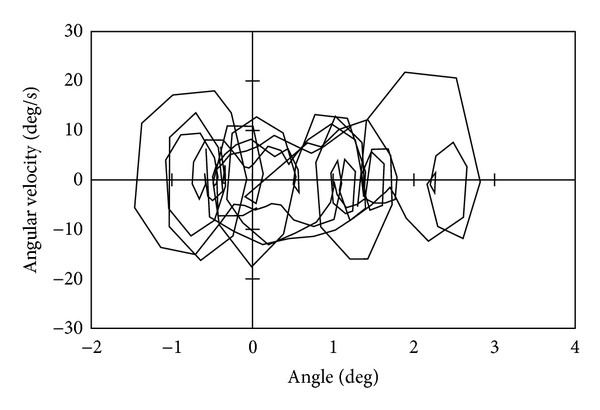
Phase portrait when walking with 30 mm breadth feet.

Figure 13 shows the phase portrait of this experiment. The phase portrait does not depict a steady closed loop compared with Figure 9. Although the robot could walk with narrow feet with 30 mm breadth, we can find that the walking stability became worse. The robot could not walk with narrower breadth feet than 30 mm.

### 3.2. Walking Experiments with a Human-Like Foot Mechanism

**Figure 14:**
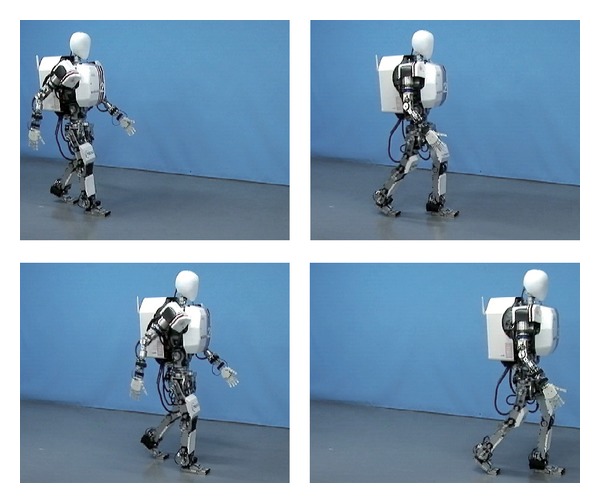
Waking experiments with the human-like foot mechanism. The walking cycle is 1.0 s/step and the step length is 450 mm/step.

Secondly, the human-like foot mechanism was attached to WABAN-2R, and forward walking experiments were conducted with a step length of 450 mm/step and a walking cycle of 1.0 s/step. In this experiment, WABIAN-2R walked with the knee-stretched, heel-contact, and toe-off motion as shown in Figure 14. WABIAN-2R tends to fall to the inside because the medial longitudinal arch of the human-like foot mechanism is more elastic than the lateral longitudinal arch. Therefore, the proposed control works well in this situation.

**Figure 15:**
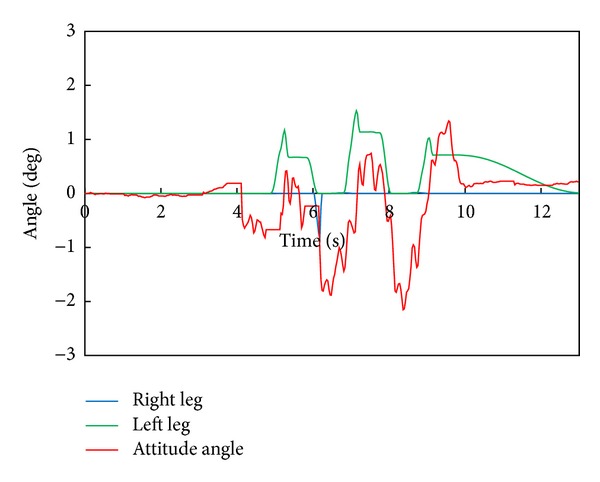
WABIAN-2R's attitude angle and the modification angle of a swing foot about the roll axis with the human-like foot mechanism.

**Figure 16:**
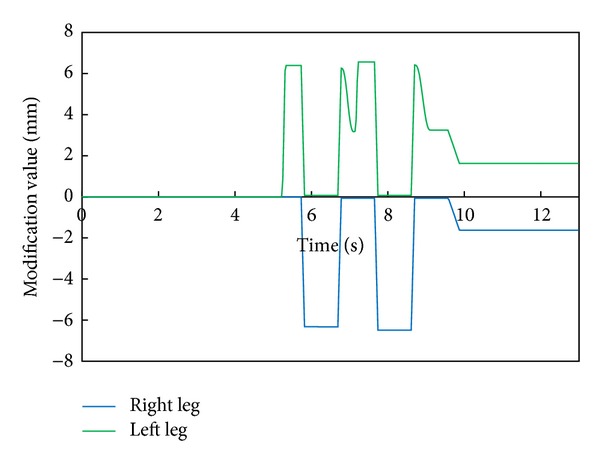
Modification value of a swing foot along the lateral axis with the human-like foot mechanism.

Figure 15 depicts the robot's attitude angle and the modification angle of a swing foot about the roll axis.  Figure 16 shows the modification value of a swing foot along the lateral axis. WABIAN-2R realized a knee-stretched walking with narrow feet by using the proposed method.

## 4. Conclusions and Future Work

We have developed a walking stabilization control for a biped humanoid robot with narrow feet. There are some previous researches on biped stabilization by changing foot placement [14, 15], but most of them assume that the foot contacts the ground at a point. Therefore, conventional stabilization controls can work well only if robot's feet contact the ground at a point, but this assumption is not acceptable for most humanoid robots [4–9] which have larger feet than human beings. The effect of areas on the feet must be considered to realize a dynamic walk.

The proposed control modifies a foot placement according to the robot's attitude angle. If a robot tends to fall down, a foot angle is modified about the roll axis so that a swing foot contacts the ground horizontally. And a foot-landing point is also changed laterally to inhibit the robot from falling to the outside. To reduce a foot-landing impact, a virtual compliance control is applied to the vertical axis and the roll and pitch axes of the foot. After foot landing on the ground, modification values of the landing foot return to zero along the vertical axis and about the roll and pitch axes. A waist trajectory is modified by changing both foot positions during double support phase. During the first half of a swing phase, a swing leg trajectory along the lateral axis returns to the preset walking pattern. Verification of the proposed method is conducted through experiments with a biped humanoid robot WABIAN-2R. WABIAN-2R realized a knee-bended walking with 30 mm breadth feet. We also confirmed that the phase portrait depicts a closed curve thanks to the proposed controller, which means a stable walk can be maintained. Moreover, WABIAN-2R mounted on a human-like foot mechanism realized a stable walking with the knee-stretched, heel-contact, and toe-off motion.

But the proposed control cannot deal with a robot's outward falling. Our next goal is to develop more robust stabilization controls to deal with the problem.
